# Genomic Portrait of Guangdong Liannan Yao Population Based on 15 Autosomal STRs and 19 Y-STRs

**DOI:** 10.1038/s41598-018-36262-x

**Published:** 2019-02-14

**Authors:** Yaoqi Liao, Ling Chen, Runze Huang, Weibin Wu, Dayu Liu, Huilin Sun

**Affiliations:** 1 Department of Endocrinology, The First Affiliated Hospital of Guangdong Pharmaceutical University, Guangzhou, 510515 China; 2 School of Forensic Medicine, Southern Medical University, Guangzhou, 510515 China

## Abstract

Here we studied the genetic polymorphism and evolutionary differentiation of the Guangdong Liannan Yao population based on 15 autosomal STR loci and 19 Y chromosomal STR loci. The blood card DNA of 302 unrelated individuals from the Yao Autonomous County of Liannan was directly amplified using an Expressmarker 16 + 19Y kit and genotyped using a 3500XL Genetic Analyzer. For the autosomal STR loci, the CPD value was over 0.999 999 999 999, while the CPE value was over 0.9999. The population comparison revealed a closer relationship between the Liannan Yao population and the She ethnic population than other reported Chinese populations. For the Y-STRs, a total of 102 unique haplotypes were obtained, 87 of which were observed only once. Both R_ST_ pairwise analysis and a multidimensional scaling plot showed that the Liannan Yao population is closely related to the Fujian She ethnic population and is significantly different from other Chinese ethnic populations. The results show that the 15 autosomal STR and 19 Y-STR loci are valuable for forensic applications and human genetic studies in the Liannan Yao population.

## Introduction

The Yao ethnic group in China is primarily distributed across the Guizhou, Hunan, Yunnan, Guangdong, and Guangxi provinces. In 2010, the population of this group consisted of 2, 796, 003 individuals (2010 census, www.stats.gov.cn). According to differences in the language, dress, customs, and living environment of the Yao population, the Yao group can be divided into Pan Yao, Guo-Shan Yao, Gaoshan Yao, Pingdi Yao, Landian Yao, Eight-Row Yao, Chashan Yao, and Baiku Yao^1^. The Yao Autonomous County of Liannan, located in Guangdong province, is home to the world’s only Eight-Row Yao population^2^. Liannan Yao has a small population of about 80,000. The Yao Autonomous County of Liannan is within northwest Guangdong province. According to folklore and historical records^3,4^, its inhabitants mainly came from the area of the Xiangjiang River, the middle and lower reaches of the Yuanjiang River, and Dongting Lake in Hunan. During the Sui-Tang Dynasties, their ancestors migrated into the Liannan Mountain district via Chenzhou and Daozhou and settled there. Genetic polymorphisms of Guangdong Liannan Yao have not yet been reported. Guangdong Liannan Yao is a special ethnic population in Guangdong in which the incidence of some diseases such as diabetes differs from those in the Guangdong Han group^5^. Through the present study, we aimed to gather autosomal STR and Y-STR genetic data for the Guangdong Liannan Yao population. Moreover, we hoped to reveal the relationship between the Guangdong Liannan Yao population and other populations.

Autosomal STR and Y-STR have been widely used in forensic evidence examinations, historical investigations, and genealogical research worldwide^6^. Autosomal STR loci have also been used to uncover the population’s genetic backdrop and structure^7^. Here we used an Expressmarker 16 + 19Y kit to detect 302 samples collected from unrelated Liannan Yao individuals (122 males and 180 females) and analyze the genetic polymorphism of 15 autosomal STR loci and 19 Y chromosome STR loci as well as assess the efficiency of this kit in the individual identification and paternity testing for the studied population. The Expressmarker 16 + 19Y kit comprises all 13 core Combined DNA Index (CODIS) STR loci (CSF1PO, FGA, TH01, TPOX, vWA, D3S1358, D5S818, D7S820, D8S1179, D13S317, D16S539, D18S51, D21S11), Amelogenin, two non-CODIS STR loci (D19S433, D2S1338) and 19 widely used Y-STR loci (DYS635, DYS456, DYS385a/b, DYS437, DYS458, DYS389I, DYS392, DYS439, DYS390, DYS393, DYS391, DYS438, DYS448, Y-GATA-H4, DYS19, DYS389II, DYS527a/b), among which, DYS385a/b and DYS527a/b are the double copy loci.

## Materials and Methods

### Sample collection

Bloodstains of 302 unrelated Liannan Yao individuals (122 males, 180 females) in Liannan Yao Autonomous County were collected. All participants were interviewed to confirm their ethnic origins and sign the Informed consents. The study was carried out in accordance with the relevant guidelines and regulations and was approved by the Ethics Committee of the First Affiliated Hospital of Guangdong Pharmaceutical University, Medical ethics review [2016] (No. 76).

### Polymerase chain reaction amplification and STR typing

Samples from the filter paper were punched using a 1.2-mm BSD600-DUET stiletto instrument (BSD, Australia) and placed into 96-well plates for direct polymerase chain reaction (PCR) amplification using an Expressmarker 16 + 19Y kit (AGCU ScienTech Incorporation, China). The kit’s details are shown in Table S1.

PCR amplification was performed using an Applied Biosystems Gene Amp PCR System 9700 thermal cycler. The reaction was performed in a 25 μL volume system containing 1X Reaction Mix (including 2.5 mM magnesium ion, 0.25 mM dNTPs, 10 mM Tris-HCl, 50 mM KCl, etc.), 1X EX16 + 19Y primer set, 2U hotstart C-Taq DNA polymerase, 1.2 mm filter paper of genomic DNA, and sdH_2_O supplying the rest of the volume. The PCR amplification cycling parameters of Expressmarker 16 + 19Y were as follows: 95 °C for 2 min; 15 cycles of 94 °C for 10 s, 60 °C for 40 s, and 72 °C for 1 min and 15 cycles of 90 °C for 10 s, 59 °C for 1 min, and 65 °C for 80 s; final extension at 60 °C for 20 min; and a final hold at 4 °C.

The PCR products were separated on a 3500XL Genetic Analyzer (Life Technologies, USA). Samples were prepared by adding 1 μL of PCR product (amplicons or allele ladder) to 12 μL of deionized formamide and 0.25 μL of marker (SIZ-500 internal lane standard). The mixture was denatured by heating 95 °C for 3 min followed by quick chilling on ice for 3 min. Standard run parameters included sample injection for 28 s at 1.2 kV. The sample DNA was genotyped using GeneMapper-ID-X software version 1.3 (Life Technologies) with the peak amplitude threshold set at 150 relative fluorescent units.

### Quality control

The study was conducted following the recommendations of DNA Commission of the International Society for Forensic Genetics (ISFG) as described by Carracedo *et al*.^8^ on DNA polymorphism analysis.

### Data analysis

For autosomal STR loci, we used the PowerStatsV12.xls software^9^ (https://www.promega.com.cn/products/genetic-identity/) to calculate the allelic frequencies, discrimination power (DP), power of exclusion, polymorphism information content (PIC), and typical paternity index. The p-values of the exact test of Hardy-Weinberg’s equilibrium, expected heterozygosity, and observed heterozygosity were calculated using Genepop software^10^ (http://genepop.curtin.edu.au/). Pairwise genetic distance (F_ST_) and p-values were calculated for each locus between populations using Arlequin V3.5 software^11^. Further, a phylogenetic tree and a principal component analysis (PCA) plot showing the interpopulation relationship were constructed using Poptree 2^12^ and Past 3.11^13^ software, respectively, according to the allelic frequency data of Liannan Yao population and 8 other reported populations (Guangxi Yao^14^, Jiangxi Han^15^, Sichuan Han^16^, Fujian Han^17^, Hunan Han^18^, Fujian She^19^, Guangdong Han^20^, and Hebei Han^21^ populations).

For the Y-STR loci, the allelic frequencies of each 19 Y-STR loci were calculated by the direct counting method. Haplotype frequencies were calculated using Arlequin SoftwareV3.5^11^. The genetic diversity or haplotype diversity was calculated as $GD/HD=n(1-{\sum Pi}^{2})/({\rm{n}}-1)$ (Pi indicates the frequency of the ith allele or haplotype; n indicates the number of samples). The discrimination capacity (DC) of the haplotypes was calculated as $DC=m/{\rm{n}}$(m indicates the number of different haplotypes; n indicates the total number of samples). Analysis of molecular variance (AMOVA) and multidimensional scaling (MDS) tests were conducted with the online tools in YHRD (http://www.yhrd.org), and our population data were compared to those of other Chinese reported populations including Chinese Han populations(Guangdong Han^22^, Shandong Han^23^, Hunan Han^24^, Guangxi Han [YA004218], and HenanHan^25–27^), the She ethnic population from Fujian (YA004031), and the Yao ethnic population from Guangxi (YA004221). A neighbor-joining phylogenetic tree based on R_ST_ values was built using MEGA 6.0 software^28^.

## Results and Discussion

### Allelic frequencies and forensic parameters of 15 autosomal STR in the Liannan Yao population

The allelic frequencies and forensic parameters of the Liannan Yao population on the basis of the 15 autosomal STR loci are shown in Table S2 and Figure S1. An STR locus can be considered highly polymorphic when its DP value is >0.80 or its power of exclusion value is >0.50^29,30^. The results of this study showed that most of the examined loci were highly polymorphic. The examined autosomal STRs had a high DP that ranged from 0.7785 (TPOX) to 0.9646 (FGA). The combined discrimination power (CPD) for the 15 loci was 0.999 999 999 999 999. The power of exclusion ranged from 0.2559 (TPOX) to 0.7293 (D8S1179), while the combined probability of excluding paternity (CPE) for the 15 loci was 0.999 995. A multiple STR amplification system can be considered as reliable for individual identification if its CPD value is over 0.999 999 999 999, and for paternity testing if its CPE value is over 0.9999. On this basis, the Expressmarker 16 + 19Y qualifies for the application of forensic individual testing and paternity testing among the Liannan Yao population.

In addition, all the STR loci were considered highly informative because they had high PIC values (PIC > 0.5 is considered highly polymorphic)^30^ that ranged from 0.5300 (TPOX) to 0.8484 (FGA). The most polymorphic and discriminatory locus in the studied population was FGA, with values of 0.8484 (PIC) and 0.9646 (DP). The highest allelic frequency (0.5629) was observed at allele 8 of the TPOX locus. The most common allele and least common allele for each locus are listed in Table S3. No significant deviations from Hardy-Weinberg expectations were observed after Bonferroni correction for any studied locus (P > 0.05).

### Interpopulation comparisons based on the 15 autosomal STRs

For autosomal STR loci, the F_ST_ distance was calculated based on the allelic frequencies and was used to compare the studied population and 8 other reported populations (Table S4). The geographical locations of the reference populations were shown in Figure S3.

Statistically significant differences (p < 0.05/15) were found between the Liannan Yao and Guangxi Yao populations at two STR loci, the Fujian She population at five loci, the Han population from Sichuan and Hunan at six STR loci, the Fujian Han and Jiangxi Han populations at seven STR loci, and the Guangdong Han and Hebei Han populations at eight STR loci. The genetic portrait of the Liannan Yao population is closer to that of the Guangxi Yao and Fujian She populations.

### Phylogenetic analyses based on the 15 autosomal STRs

**Figure 1:**
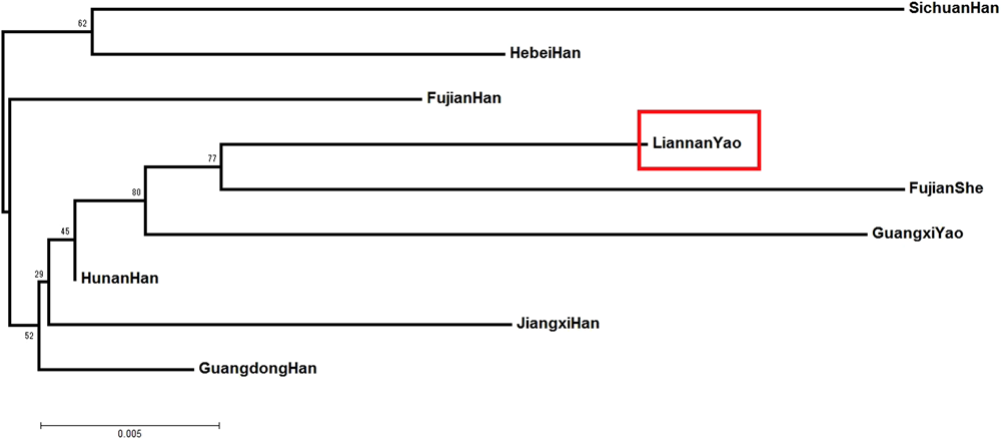
The phylogenetic tree based on allele frequencies of 15 autosomal STR loci for Lianyao population and 8 other reported populations (Guangxi Yao, Jiangxi Han, Sichuan Han, Fujian Han, Hunan Han, Fujian She, Guangdong Han, and Hebei Han populations).

The neighbor-joining phylogenetic tree (Fig. 1) mirrors the historical and geographical backgrounds of the studied population. The phylogenetic tree showed that the Liannan Yao and Fujian She populations were clustered in one branch, while the other Chinese populations were clustered in another branch. The Liannan Yao population in this study was closest to the Fujian She ethnic group, followed by the Guangxi Yao and Hunan Han populations.

Historically, the Yao and She ethnic groups originated from the same ancient ethnic group. These ancient peoples migrated from Xiangnan district, currently known as Hunan province. Some of them moved eastward to the junction of Jiangxi, Guangdong, and Fujian provinces and evolved into the She ethnic groups, whereas the others moved into northern Guangxi and Guangdong and evolved into the Yao ethnic groups. Later, the She ethnic groups extended to eastern Fujian and southern Zhejiang, while the Yao people extended to the west and south of Guangxi and Guangdong. Eventually, the overall distribution pattern of the present Yao and She ethnic groups was formed. Our study results according to the phylogenetic analyses based on the 15 autosomal STRs support the migration history of the two populations. Moreover, these results supported that the Fujian She population and Guangdong Liannan Yao population have a remote geographic distance but a limited genetic distance.

### PCA based on the 15 autosomal STRs

**Figure 2:**
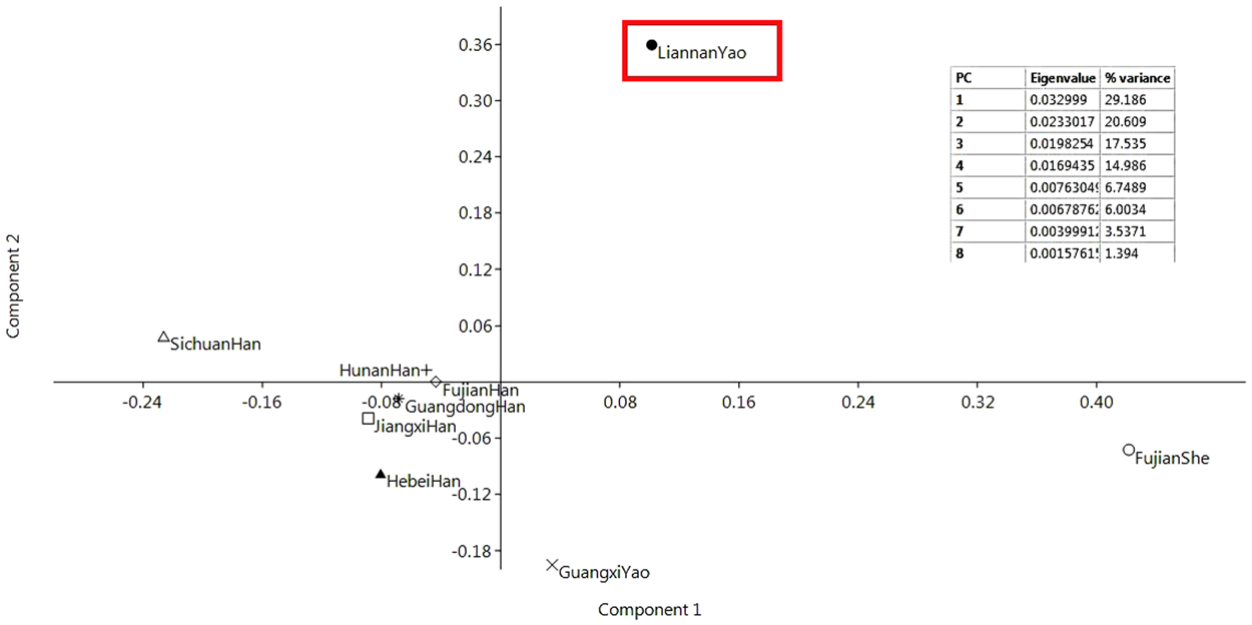
Principal component analysis plot based on allele frequencies of 15 autosomal STR loci for the Liannan Yao population and 8 other reported populations (Guangxi Yao, Jiangxi Han, Sichuan Han, Fujian Han, Hunan Han, Fujian She, Guangdong Han, and Hebei Han populations).

A PCA plot was drawn based on the allelic frequencies of 15 STRs (Fig. 2). The Liannan Yao, Guangxi Yao, and Fujian She populations were clustered in the right side; the Sichuan Han population was clustered in the left upper quadrant; and the Chinese Han populations from Hebei, Hunan, Guangdong, Jiangxi and Fujian province were clustered lower near to the center.

As described above, the Yao and She ethnic groups share the same origin. The genetic distance between the Liannan Yao and Fujian She populations is the closest, followed by the Guangxi Yao and Hunan Han populations.

### Haplotypic structure of 19 Y-STR loci in the Liannan Yao population

The data were submitted to the Y-STR Haplotype Reference Database (YHRD) for checking as Liannan China [Yao], and can be retrieved using the accession number YA004311 (http://www.yhrd.org). The 122 unrelated individuals were genotyped with 19 Y-STR loci, and allelic frequencies of each loci are presented in Table S5. 30 haplotypes were found for DYS385a/b, while 19 were observed for DYS527a/b. A total of 118 alleles were observed, and the corresponding allelic frequencies ranged from 0.0041 (DYS458) to 0.8770 (DYS391). The most common allele and least common allele for each locus are listed in the Table S3, as autosomal STRs before. Similarly, a total of 102 Y-STR haplotypes (Table S6) were observed, of which 87 were unique and 15 were observed for more than two individuals. The genetic diversity (Figure S2) values of the 19 loci ranged from 0.2211 (DYS391) to 0.8862 (DYS385a/b). The haplotype diversity and DC was 0.9891 and 0.8197, respectively.

### Inter-population comparisons based on the 15 shared Y-STRs

For the Y-STR loci, the studied data were compared to those of other Chinese reported populations. The geographical locations of the reference populations were shown in Figure S3. The R_ST_ values and corresponding p-values were computed using AMOVA (Table 1). The results indicate that after applying Bonferroni’s correction, R_ST_ values did not differ significantly among the Liannan Yao and Fujian She population (R_st_ = 0.0363; p > 0.0018, 28 pairs), but they differed significantly for following populations: Guangdong Han, Guangxi Han, Henan Han, Hunan Han, Shandong Han, and Guangxi Yao (p < 0.0018).

**Table 1:** Population pairwise genetic distances (R_ST_) and associated probability values (p-values) based on 19 Y-STRs between the LiannanYao population and 7 other populations (Guangdong Han, Shandong Han, Hunan Han, Guangxi Han, Henan Han, Fujian She, and Guangxi Yao).

Groups	Liannan Yao	Guangdong Han	Guangxi Han	Henan Han	Hunan Han	Shandong Han	Fujian She	Guangxi Yao
Liannan Yao	—	0.0000	0.0000	0.0000	0.0000	0.0000	0.0020	0.0000
Guangdong Han	0.0882	—	0.1480	0.0000	0.0542	0.0000	0.0004	0.0000
Guangxi Han	0.1077	0.0005	—	0.0000	0.0606	0.0000	0.0000	0.0000
Henan Han	0.1078	0.0117	0.0108	—	0.0000	0.0003	0.0001	0.0000
Hunan Han	0.0997	0.0018	0.0015	0.0078	—	0.0001	0.0009	0.0000
Shandong Han	0.0867	0.0124	0.0144	0.0034	0.0086	—	0.0008	0.0000
Fujian She	0.0363	0.0307	0.0406	0.0326	0.0292	0.0238	—	0.0022
Guangxi Yao	0.0812	0.0429	0.0502	0.0426	0.0418	0.0363	0.0352	—

p-values are shown above the diagonal line and R_ST_ values are shown below it.

### Phylogenetic analyses based on the 15 shared Y-STRs

**Figure 3:**
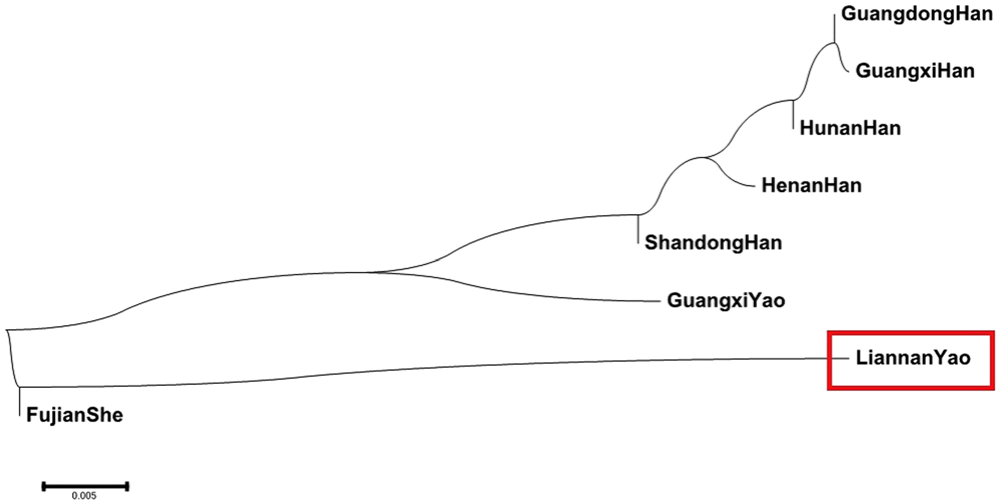
A Neighbor-Joining tree based on 19 Y-STRs showing the relationship between Liannan Yao group and other 7 populations (Guangdong Han, Shandong Han, Hunan Han, Guangxi Han, Henan Han, Fujian She, and Guangxi Yao populations).

**Figure 4:**
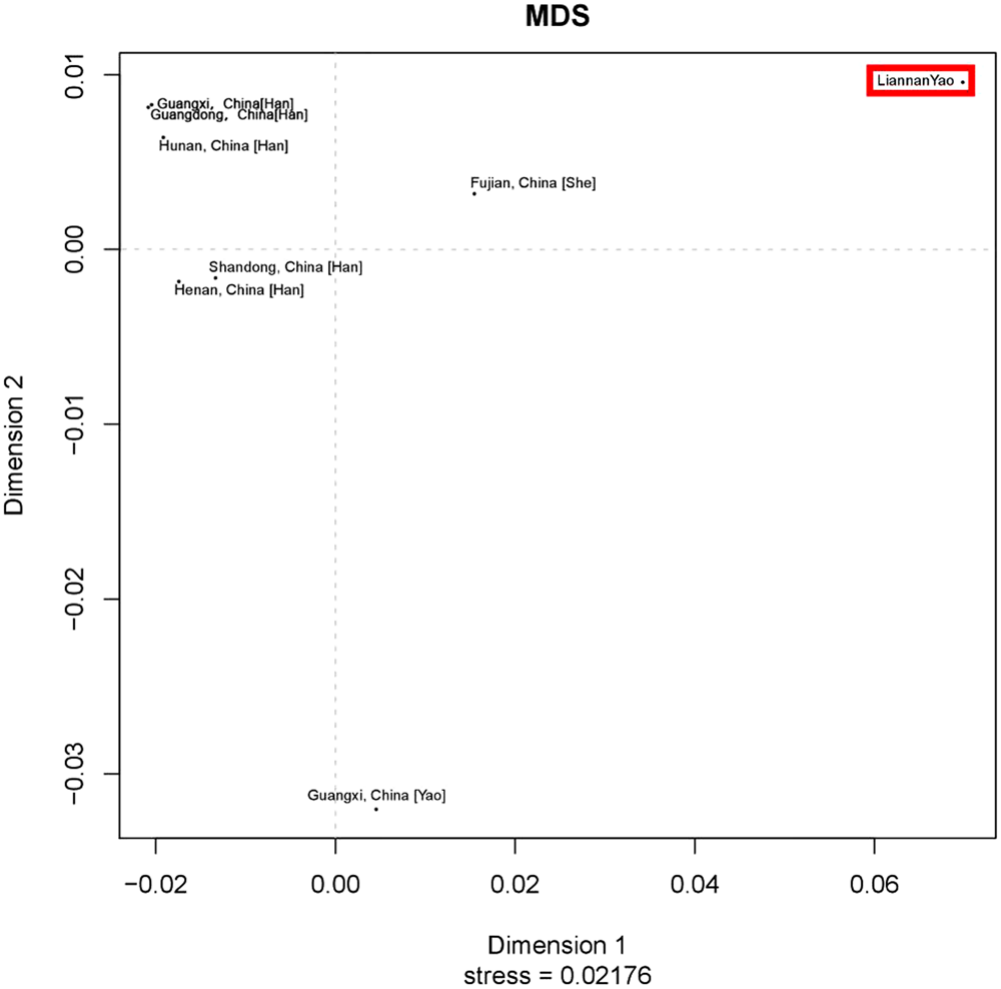
A MDS plot based on 19 Y-STRs between Lianyao population and other 7 populations (Guangdong Han, Shandong Han, Hunan Han, Guangxi Han, Henan Han, Fujian She, and Guangxi Yao).

In the neighbor-joining tree, the Liannan Yao population first clustered with the Fujian She ethnic population, second with the Guangxi Yao ethnic population, and third with the Guangdong Han and Hunan Han populations (Fig. 3). As shown in the MDS plot, there were clear differences between the Liannan Yao and 7 other populations (Fig. 4). Moreover, compared with other Chinese Han populations, the Liannan Yao population was closer to the Fujian She population, followed by the Guangxi Yao population. This shows Liannan Yao population has a close genetic distance with the She and other Yao ethnic populations, which support the historian’s perspective that the Liannan Yao and Fujian She ethnic populations share the same origin.

There was one potential limitation in the present study that the included groups for population comparison between autosomal STR and Y-STR are different, due to the limited available relevant data. However, these analyses all indicate that Liannan Yao population has a close relationship with Fujian She and Guangdong Yao population, also support the migration history of the two populations that Yao and She have the same origin. Moreover, there are clear differences between the Liannan Yao and other Chinese Han population but Guangdong Han population. This result might indicate that the Liannan Yao integrated gradually with natives, such as Guangdong Han population, following its geographical migration, which is also corresponded with the historical records^3,4^.

## Conclusion

These results demonstrate that the Liannan Yao population is an independent exclusive ethnicity and has some unique genetic characteristics. 15 autosomal STR and 19 Y-STR loci for Liannan Yao population are informative, and Expressmarker 16 + 19Y kit is suitable for personal identification and paternity testing within this population. These data could be helpful for inferring the genetic genealogy evolution and ancient human migration patterns of the Liannan Yao population.

## Electronic supplementary material

Figure S1

Figure S2

Figure S3

Table S1

Table S2

Table S3

Table S4

Table S5

Table S6
