# Supplementary material for: Genomic Portrait of Guangdong Liannan Yao Population Based on 15 Autosomal STRs and 19 Y-STRs
Source: Sci Rep. 2019 Feb 14;9:2141. doi: 10.1038/s41598-018-36262-x (PMC6376128; doi:10.1038/s41598-018-36262-x)
Supplement: Supplementary file 4 — Table S1 [file 41598_2018_36262_MOESM4_ESM.pdf]

Genomic Portrait of Guangdong Liannan Yao Population Based on 15 Autosomal STRs and 19 Y-STRs

Yaoqi Liao[1], Ling Chen[2], Runze Huang[1], Weibin Wu[2], Dayu Liu[2], Huilin Sun[1] *

[1] Department of Endocrinology, The First Affiliated Hospital of Guangdong Pharmaceutical University, 510515, China.

[2] School of Forensic Medicine, Southern Medical University, Guangzhou, 510515, China.

Table S1. The infomation of STR loci contained in Expressmarker 16+19Y kit.

Locus	fluorescent dye label	EX16+19Y Allelic Ladder
D3S1358	FAM	12,13,14,15,16,17,18,19
D13S317	FAM	6,8,9,10,11,12,13,14,15
D7S820	FAM	6,7,8,9,10,11,12,13,14,15
D16S539	FAM	5,6,8,9,10,11,12,13,14,15
TPOX	HEX	6,7,8,9,10,11,12,13
TH01	HEX	4,5,6,7,8,9,9.3,10,11
D2S1338	HEX	15,16,17,18,19,20,21,22,23,24,25,26
CSF1PO	HEX	7,8,9,10,11,12,13,14,15
D19S433	TAMRA	9,10,11,12,12.2,13,13.2,14,14.2,15,15.2,16,16.2,17,17.2

Locus	fluorescent dye label	EX16+19Y Allelic Ladder
vWA	TAMRA	14,15,16,17,18,19,20,21
D18S51	TAMRA	10,11,12,13,14,15,16,17,18,19,20,21,22,23,24,25
D8S1179	ROX	8,9,10,11,12,13,14,15,16,17,18,19
D5S818	ROX	7,8,9,10,11,12,13,14,15
D21S11	ROX	25,26,27,28,28.2,29,29.2,30,30.2,31,31.2,32,32.2,33,33.2,34,34.2,35,35.2,36,37
FGA	ROX	17,18,19,20,21,22,23,24,25,26,27,28,29,30
Amelogenin	ROX	X,Y
DYS635	ROX	19,20,21,22,23,24,25
DYS393	VIG	12,13,14,15,16
DYS389I	VIG	11,12,13,14,15
DYS439	VIG	7,8,10,11,12,13,14,15
DYS389II	VIG	26,27,28,29,30,31,32,33
DYS527a/b	VIG	17,18,19,20,21,22,23,24,25,26,27,28
Y_GATA_H4	VIG	9,10,11,12,13,14

**Locus**	**fluorescent dye label**	**EX16+19Y Allelic Ladder**
DYS391	FAM	6,7,8,9,10,11,12,13
DYS392	FAM	7,8,9,10,11,12,13,14,15,16,17
DYS448	FAM	17,18,19,20,21,22,23
DYS438	FAM	8,9,10,11,12,13
DYS456	HEX	13,14,15,16,17,18
DYS385a/b	HEX	10,11,12,13,14,15,16,17,18,19,20,21,22,23,24
DYS458	HEX	12,13,14,15,16,17,18,19,20,21,22
DYS437	TAMRA	12,13,14,15,16,17
DYS19	TAMRA	12,13,14,15,16,17,18
DYS390	TAMRA	19,20,21,22,23,24,25,26
