# Supplementary material for: Genomic Portrait of Guangdong Liannan Yao Population Based on 15 Autosomal STRs and 19 Y-STRs
Source: Sci Rep. 2019 Feb 14;9:2141. doi: 10.1038/s41598-018-36262-x (PMC6376128; doi:10.1038/s41598-018-36262-x)
Supplement: Supplementary file 5 — Table S2 [file 41598_2018_36262_MOESM5_ESM.pdf]

Genomic Portrait of Guangdong Liannan Yao Population Based on 15 Autosomal STRs and 19 Y-STRs

Yaoqi Liao[1], Ling Chen[2], Runze Huang[1], Weibin Wu[2], Dayu Liu[2], Huilin Sun[1] *

[1] Department of Endocrinology, The First Affiliated Hospital of Guangdong Pharmaceutical University, 510515, China.

[2] School of Forensic Medicine, Southern Medical University, Guangzhou, 510515, China.

Table S2. Allele frequency distribution of 15 autosomal STRs in Liannan Yao population (n=302)

Allele	D3S1358	D13S317	D7S820	D16S539	TPOX	TH01	D2S1338	D8S1179	D19S433	vWA	D5S818	FGA	D21S11	D18S51	CSF1PO
4	-	-	-	-	-	0.0017	-	-	-	-	-	-	-	-	-
6	-	-	-	-	-	0.1346	-	-	-	-	0.0017	-	-	-	-
7	-	-	0.0017	-	-	0.2757	-	-	-	-	0.0447	-	-	-	0.0265
8	-	0.3344	0.0811	0.0017	0.5629	0.0548	-	-	-	-	-	-	-	-	-
9	-	0.1474	0.0298	0.2086	0.0480	0.4419	-	-	-	-	0.0646	-	-	-	0.0149
9.1	-	-	0.0099	-	-	-	-	-	-	-	-	-	-	-	-
9.3	-	-	-	-	-	0.0316	-	-	-	-	-	-	-	-	-
10	--	0.1391	0.1921	0.1623	0.0381	0.0598	-	0.1990	-	-	0.2666	-	-	-	0.1755
11	-	0.2252	0.3891	0.2368	0.2930	-	-	0.0901	0.0017	-	0.3195	-	-	-	0.1766

Allele	D3S1358	D13S317	D7S820	D16S539	TPOX	TH01	D2S1338	D8S1179	D19S433	vWA	D5S818	FGA	D21S11	D18S51	CSF1PO
12	-	0.1258	0.2781	0.2401	0.0579	-	-	0.0816	0.0315	-	0.1755	-	-	0.0894	0.4901
13	-	0.0281	0.0182	0.1391	-	-	-	0.1905	0.3262	-	0.1175	-	-	0.1043	0.1175
13.2	-	-	-	-	-	-	-	-	0.0447	-	--	-	-	-	-
14	0.0199	-	-	0.0116	-	-	-	0.1701	0.1738	0.3212	0.0083	-	-	0.3427	0.0033
14.2	-	-	-	-	-	-	-	-	0.1159	-	-	-	-	-	-
15	0.2649	-	-	-	-	-	-	0.1650	0.0430	0.0430	0.0017	-	-	0.1407	-
15.2	-	-	-	-	-	-	-	-	0.2152	-	-	-	-	-	-
16	0.2997	-	-	-	-	-	0.0050	0.0986	0.0149	0.1474	-	0.0017	-	0.1407	-
16.2	-	-	-	-	-	-	-	-	0.0331	-	-	-	-	-	-
17	0.3510	-	-	-	-	-	0.0464	0.0017	-	0.1821	-	-	-	0.0381	-
18	0.0613	-	-	-	-	-	0.0513	0.0034	-	0.2103	-	0.0066	-	0.0679	-
19	0.0033	-	-	-	-	-	0.1275	-	-	0.0778	-	0.0629	-	0.0248	-
20	-	-	-	-	-	-	0.0844	-	-	0.0182	-	0.0182	-	0.0116	-
20.2	-	-	-	-	-	-	-	-	-	-	-	0.0017	-	-	-

Allele	D3S1358	D13S317	D7S820	D16S539	TPOX	TH01	D2S1338	D8S1179	D19S433	vWA	D5S818	FGA	D21S11	D18S51	CSF1PO
21	-	-	-	-	-	-	0.0099	-	-	-	-	0.1738	-	0.0066	-
22	-	-	-	-	-	-	0.1258	-	-	-	-	0.1474	-	0.0232	-
22.2	-	-	-	-	-	-	-	-	-	-	-	0.0017	-	-	-
23	-	-	-	-	-	-	0.2798	-	-	-	-	0.1573	-	0.0099	-
23.2	-	-	-	-	-	-	-	-	-	-	-	0.0166	-	-	-
24	-	-	-	-	-	-	0.1904	-	-	-	-	0.1821	-	-	-
24.2	-	-	-	-	-	-	-	-	-	-	-	0.0315	-	-	-
25	-	-	-	-	-	-	0.0728	-	-	-	-	0.1358	-	-	-
25.2	-	-	-	-	-	-	-	-	-	-	-	0.0083	-	-	-
26	-	-	-	-	-	-	0.0066	-	-	-	-	0.0497	-	-	-
27	-	-	-	-	-	-	-	-	-	-	-	0.0033	0.0132	-	-
28	-	-	-	-	-	-	-	-	-	-	-	0.0017	0.0712	-	-
28.2	-	-	-	-	-	-	-	-	-	-	-	-	0.0017	-	-
29	-	-	-	-	-	-	-	-	-	-	-	-	0.1805	-	-

Allele	D3S1358	D13S317	D7S820	D16S539	TPOX	TH01	D2S1338	D8S1179	D19S433	vWA	D5S818	FGA	D21S11	D18S51	CSF1PO
30	-	-	-	-	-	-	-	-	-	-	-	-	0.2202	-	-
31.2	-	-	-	-	-	-	-	-	-	-	-	-	0.1010	-	-
32	-	-	-	-	-	-	-	-	-	-	-	-	0.0182	-	-
32.2	-	-	-	-	-	-	-	-	-	-	-	-	0.1987	-	-
33	-	-	-	-	-	-	-	-	-	-	-	-	0.0017	-	-
33.2	-	-	-	-	-	-	-	-	-	-	-	-	0.0977	-	-
34.2	-	-	-	-	-	-	-	-	-	-	-	-	0.0017	-	-
35													0.0017		
