# Supplementary material for: Genomic Portrait of Guangdong Liannan Yao Population Based on 15 Autosomal STRs and 19 Y-STRs
Source: Sci Rep. 2019 Feb 14;9:2141. doi: 10.1038/s41598-018-36262-x (PMC6376128; doi:10.1038/s41598-018-36262-x)
Supplement: Supplementary file 6 — Table S3 [file 41598_2018_36262_MOESM6_ESM.pdf]

Genomic Portrait of Guangdong Liannan Yao Population Based on 15 Autosomal STRs and 19 Y-STRs

Yaoqi Liao[1], Ling Chen[2], Runze Huang[1], Weibin Wu[2], Dayu Liu[2], Huilin Sun[1] *

[1] Department of Endocrinology, The First Affiliated Hospital of Guangdong Pharmaceutical University, 510515, China.

[2] School of Forensic Medicine, Southern Medical University, Guangzhou, 510515, China.

Table S3. MCA, LCA and their total number at each locus (MCA- Most Common Allele, LCA- Least Common Allele).

S. No.	Locus	MCA	LCA	Total no. of observed allele per locus
1	D3S1358	17	19	6
2	D13S317	8	13	6
3	D7S820	11	7	8
4	D16S539	12	8	7
5	TPOX	8	10	5
6	THO1	9	4	7
7	D2S1338	23	16	11
8	D8S1179	10	17	9
9	FGA	24	16, 20.2, 22.2, 28	17
10	D19S433	13	11	10
11	vWA	14	20	7
12	D21S11	30	28.2, 33, 34.2, 35	13
13	D18S51	14	21	12
14	D5S818	11	6, 15	9

S. No.	Locus	MCA	LCA	Total no. of observed allele per locus
15	CSF1PO	12	14	7
16	DYS391	10	9	3
17	DYS392	13	15	5
18	DYS448	20	21	4
19	DYS438	10	9	3
20	DYS456	15	17	5
21	DYS458	17	14	8
22	DYS437	15	14	2
23	DYS393	12	9,15	5
24	DYS19	16	17	4
25	DYS439	12	14	5
26	DYS635	21	25	7
27	DYS389I	12	11	4
28	DYS389II	28	31	5
29	DYS390	24	22	5
30	YGATAH4	12	10	4
**S. No.**	**Locus**	**Most Common Haplotype**	**Least Common Haplotype**	**Total number of observed haplotype per locus**
31	DYS385a/b	13/20	11/12，11/14，11/16，11/20，12/13，12/18，12/19，12/21，12/22，13/13，13/16，13/23，14/17，14/18，15/21，18/18	30
32	DYS527a/b	20/23	20/21，20/27，21/21，23/27，24/24	19
