# Supplementary material for: Genomic Portrait of Guangdong Liannan Yao Population Based on 15 Autosomal STRs and 19 Y-STRs
Source: Sci Rep. 2019 Feb 14;9:2141. doi: 10.1038/s41598-018-36262-x (PMC6376128; doi:10.1038/s41598-018-36262-x)
Supplement: Supplementary file 7 — Table S4 [file 41598_2018_36262_MOESM7_ESM.pdf]

Genomic Portrait of Guangdong Liannan Yao Population Based on 15 Autosomal STRs and 19 Y-STRs

Yaoqi Liao[1], Ling Chen[2], Runze Huang[1], Weibin Wu[2], Dayu Liu[2], Huilin Sun[1] *

[1] Department of Endocrinology, The First Affiliated Hospital of Guangdong Pharmaceutical University, 510515, China.

[2] School of Forensic Medicine, Southern Medical University, Guangzhou, 510515, China.

Table S4. Pairwise genetic distance (F_{ST}) and p values of 15 autosomal STRs for Liannan Yao population and other 8 populations

*: Significant differentiation test p-values, after Bonferroni correction.

Loci	Index	Fujian Han	Guangdong Han	Jiangxi Han	Hunan Han	Fujian She	Sichuan Han	Hebei Han	Guangxi Yao
FGA	F_{st}	0.0095	0.0041	0.0038	0.0048	0.0097	0.0037	0.0072	-0.0011
	p	0.0000*	0.0181	0.0561	0.0181	0.0000*	0.0181	0.0000*	0.5912
TH01	F_{st}	0.0014	0.0022	0.0016	-0.0004	0.0143	0.0030	0.0093	0.0092
	p	0.3005	0.1172	0.1865	0.4598	0.0362	0.0689	0.0508	0.1023
TPOX	F_{st}	0.0063	0.0050	0.0025	0.0032	-0.0002	0.0044	0.0085	0.0033
	p	0.0742	0.0508	0.1892	0.1308	0.3228	0.0508	0.0464	0.2563

**Loci**	**Index**	**Fujian Han**	**Guangdong Han**	**Jiangxi Han**	**Hunan Han**	**Fujian She**	**Sichuan Han**	**Hebei Han**	**Guangxi Yao**
D3S1358	F_{st}	0.0250	0.0132	0.0076	0.01016	0.0343	0.0119	0.0295	0.0069
	p	0.0000*	0.0000*	0.0409	0.0000*	0.0000*	0.0000*	0.0000*	0.1719
D13S317	F_{st}	0.0012	0.0017	-0.0023	-0.0017	0.0066	-0.0007	-0.0005	-0.0045
	p	0.3825	0.1570	0.9203	0.9124	0.0561	0.7541	0.5346	0.7714
D7S820	F_{st}	0.0020	0.0058	0.0087	0.0031	0.0216	0.0061	0.0096	0.0033
	p	0.1365	0.0000*	0.0000*	0.0948	0.0000*	0.0181	0.0508	0.2361
D16S539	F_{st}	0.0028	0.0016	0.0009	0.0045	0.0008	0.0045	0.0110	0.0155
	p	0.1335	0.1719	0.2635	0.0181	0.5012	0.0181	0.0000*	0.0409
D2S1338	F_{st}	0.01000	0.0106	0.0078	0.0096	0.0334	0.0080	0.0088	0.0267
	p	0.0000*	0.0000*	0.0000*	0.0000*	0.0000*	0.0000*	0.0000*	0.0000*
CSF1PO	F_{st}	0.0500	0.0109	0.0301	0.0111	0.0099	0.0105	0.0093	0.0222
	p	0.0825	0.0000	0.0000*	0.0000*	0.0362	0.0000*	0.0181	0.0301

Loci	Index	Fujian Han	Guangdong Han	Jiangxi Han	Hunan Han	Fujian She	Sichuan Han	Hebei Han	Guangxi Yao
D19S433	F_{st}	0.0049	0.0070	0.0294	0.0040	0.0020	0.0042	0.0069	-0.0003
	p	0.0301	0.0000*	0.0000*	0.0654	0.1829	0.0301	0.0464	0.4594
vWA	F_{st}	0.0098	0.0040	0.0007	0.0044	-0.0028	0.0019	0.0053	-0.0049
	p	0.0000*	0.0464	0.3306	0.0867	0.8569	0.1204	0.0508	0.8834
D18S51	F_{st}	0.0187	0.0177	0.0166	0.0145	0.0043	0.0160	0.0120	0.0142
	p	0.0000*	0.0000*	0.0000*	0.0000*	0.1023	0.0000*	0.0000*	0.0464
D8S1179	F_{st}	0.1222	0.0995	0.0961	0.1095	0.1325	0.1019	0.1109	0.1099
	p	0.0000*	0.0000*	0.0000*	0.0000*	0.0000*	0.0000*	0.0000*	0.0000*
D5S818	F_{st}	-0.0002	0.0035	0.0036	-0.0007	0.0149	0.0027	0.0120	-0.0021
	p	0.3800	0.0409	0.0783	0.6147	0.0181	0.0654	0.0000*	0.6199
D21S11	F_{st}	0.0195	0.0212	0.0080	0.0062	0.0100	0.1036	0.0139	0.0016
	p	0.0000*	0.0000*	0.0000*	0.0000*	0.0181	0.0000*	0.0000*	0.3137
