# Supplementary material for: Genomic Portrait of Guangdong Liannan Yao Population Based on 15 Autosomal STRs and 19 Y-STRs
Source: Sci Rep. 2019 Feb 14;9:2141. doi: 10.1038/s41598-018-36262-x (PMC6376128; doi:10.1038/s41598-018-36262-x)
Supplement: Supplementary file 8 — Table S5 [file 41598_2018_36262_MOESM8_ESM.pdf]

Genomic Portrait of Guangdong Liannan Yao Population Based on 15 Autosomal STRs and 19 Y-STRs

Yaoqi Liao[1], Ling Chen[2], Runze Huang[1], Weibin Wu[2], Dayu Liu[2], Huilin Sun[1] *

[1] Department of Endocrinology, The First Affiliated Hospital of Guangdong Pharmaceutical University, 510515, China.

[2] School of Forensic Medicine, Southern Medical University, Guangzhou, 510515, China.

Table S5. Allele frequency distribution of 19 Y-STRs in Liannan Yao population (n=122).

Allele	DYS391	DYS392	DYS448	DYS438	DYS456	DYS458	DYS437	DYS393	DYS19	DYS439	DYS635	DYS389I	DYS389II	DYS390	YGATAH4	haplotype	DYS385a/b	haplotype	DYS385a/b	haplotype	DYS385a/b
9	0.0164	-	-	0.0164	-	-	-	0.0082	-	-	-	-	-	-	-	11/11	0.0164	12/22	0.0082	15/19	0.0902
10	0.877	-	-	0.7787	-	-	-	-	-	0.123	-	-	-	-	0.041	11/12	0.0082	13/13	0.0082	15/20	0.0328
11	0.1066	0.041	-	0.2049	-	-	-	-	-	0.377	-	0.0082	-	-	0.1066	11/14	0.0082	13/14	0.0164	15/21	0.0082
12	-	0.1148	-	-	-	-	-	0.623	-	0.3279	-	0.7459	-	-	0.7541	11/16	0.0082	13/16	0.0082	18/18	0.0082
13	-	0.5738	-	-	0.0902	-	-	0.2787	-	0.1639	-	0.123	-	-	0.0984	11/19	0.0164	13/17	0.0820	-	-
14	-	0.2541	-	-	0.0574	0.0082	0.2459	0.082	0.2295	0.0082	-	0.123	-	-	-	11/20	0.0082	13/19	0.0984	-	-
15	-	0.0164	-	-	0.6066	0.0246	0.7541	0.0082	0.2623	-	-	-	-	-	-	12/13	0.0082	13/20	0.2869	-	-
16	-	-	-	-	0.2213	0.1639	-	-	0.4508	-	-	-	-	-	-	12/16	0.0656	13/21	0.0328	-	-
17	-	-	-	-	0.0246	0.3197	-	-	0.0574	-	-	-	-	-	-	12/17	0.0328	13/23	0.0082	-	-

Allele	DYS391	DYS392	DYS448	DYS438	DYS456	DYS458	DYS437	DYS393	DYS19	DYS439	DYS635	DYS389I	DYS389II	DYS390	YGATAH4	haplotype	DYS527a/b	haplotype	DYS527a/b	-	-
18	-	-	0.0984	-	-	0.2459	-	-	-	-	-	-	-	-	-	12/18	0.0082	14/17	0.0082	-	-
19	-	-	0.1967	-	-	0.127	-	-	-	-	0.0738	-	-	-	-	12/19	0.0082	14/18	0.0082	-	-
20	-	-	0.6148	-	-	0.082	-	-	-	-	0.1639	-	-	-	-	12/20	0.0164	14/19	0.0164	-	-
21	-	-	0.0902	-	-	0.0246	-	-	-	-	0.2623	-	-	-	-	12/21	0.0082	14/20	0.0656	-	-
22	-	-	-	-	-	-	-	-	-	-	0.1393	-	-	0.0328	-	19/20	0.0164	21/23	0.0246	-	-
23	-	-	-	-	-	-	-	-	-	-	0.1967	-	-	0.1803	-	19/21	0.0164	21/24	0.0574	-	-
24	-	-	-	-	-	-	-	-	-	-	0.1066	-	-	0.4836	-	19/22	0.0164	21/25	0.0246	-	-
25	-	-	-	-	-	-	-	-	-	-	0.0574	-	-	0.2623	-	20/21	0.0082	22/22	0.1230	-	-
26	-	-	-	-	-	-	-	-	-	-	-	-	-	0.041	-	20/23	0.3770	22/23	0.0246	-	-
27	-	-	-	-	-	-	-	-	-	-	-	-	0.1148	-	-	20/24	0.0984	22/24	0.0246	-	-
28	-	-	-	-	-	-	-	-	-	-	-	-	0.5328	-	-	20/26	0.0738	23/23	0.0246	-	-
29	-	-	-	-	-	0.0041	-	-	-	-	-	-	0.1393	-	-	20/27	0.0082	23/27	0.0082	-	-
30	-	-	-	-	-	-	-	-	-	-	-	-	0.1148	-	-	21/21	0.0082	24/24	0.0082	-	-
31	-	-	-	-	-	-	-	-	-	-	-	-	0.0984	-	-	21/22	0.0574	-	-	-	-
